# A Gut‐Centric View of Ageing: A Pilot Analysis Mapping Age‐Associated Immune and Molecular Alterations in Colonic Mucosa Using Spatial Proteomics

**DOI:** 10.1111/acel.70605

**Published:** 2026-06-29

**Authors:** Jack Sullivan, Jessica Conway, Sophie J. Hopkin, Myriam Chimen, Niharika A. Duggal

**Affiliations:** ^1^ Department of Inflammation and Ageing, School of Immunity, Inflammation and Immunology University of Birmingham Birmingham UK

## Abstract

Investigating age‐associated changes in intestine and understanding immune‐related intestinal dysfunctions is essential for promoting healthy ageing. Mucosal surfaces represent a distinct immune compartment enriched with specialised lymphocytes that interact dynamically with the epithelial layer. In this study, we present novel spatially resolved insights into the cellular and molecular alterations in the ageing murine gut mucosa. Our findings reveal a complex network of interdependent age‐related changes, including accumulation of senescent T cells near the epithelial layer, increased expression of ribosomal protein S6 kinase (mTOR target), upregulation of glycolytic enzyme (GAPDH) linked with metabolic reprogramming and elevated apoptosis activity (caspase 3). Together, these molecular signatures point to a progressive dysregulation of mucosal immune homeostasis highlighting reduced tissue resilience as we age. Furthermore, our assessment revealing increased gut‐homing of aged naïve human T cells towards the mucosa suggests that exposure to antigenic stimulation at the mucosa is driving this senescence state. Although further investigations are needed to elucidate the causal mechanisms. Our findings highlight the therapeutic potential of pharmacological drugs (e.g., metformin, senolytics) and lifestyle‐based approaches (such as caloric restriction) targeting key pathways reported to modulate the immune‐epithelial interactions and support intestinal homeostasis during ageing.

## Graphical abstract

Spatial proteomics reveals age‐associated changes in the gut mucosa. Spatial proteomics reveals interconnected molecular and immune alterations across distinct regions of the mucosal surface, including the accumulation of senescence‐associated markers adjacent to the epithelial layer, evidence of metabolic reprogramming and upregulated mTOR signalling. Within the Peyer's patch region, ageing is further characterised by differential expression of T‐cell exhaustion markers between young and aged mice, highlighting compartment‐specific immune remodelling.

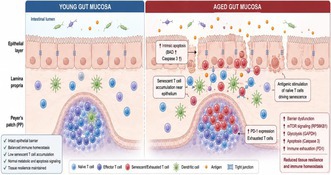

## 1 Introduction, Results and Discussion

The epithelium of the gastrointestinal (GI) tract forming the body's largest interface with the external environment encompasses endothelial, mucus layer and immunological components and limits the permeation of luminal microorganisms, antigens and endotoxins into the bloodstream (Zihni et al. 2016). Portions of the GI tract are lined by specialised gut‐associated lymphoid tissues (GALT) known as Peyer's patches (PPs) and the mucosal immune system depends on the cooperation of lymphocytes populating the epithelial barrier and within PPs. Advancing age is accompanied by physiological changes to the intestine, including mucus layer thinning and remodelling of intestinal epithelial tight junction proteins, such as occludin, that contribute towards the breakdown of intestinal barrier function permitting microbe or pathogen‐associated molecular patterns (MAMPs or PAMPs), such as lipopolysaccharide (LPS), translocation from the gut lumen enter into the bloodstream (Salazar et al. 2023) which has been linked to triggering inflammation (Thevaranjan et al. 2017), frailty and declining health in aged hosts (Stehle Jr. et al. 2012).

Concurrent with changes in intestinal homeostasis, we also observe age‐associated remodelling of the immune system and a decline in immune competence, termed immunesenescence (Duggal 2018). A hallmark of human immunesenescence is the accumulation of late‐differentiated memory T cells (CD28^−ve^) with features of replicative senescence, such as genotoxic damage, and expansion of regulatory T cells (Mittlebrunn and Kroemer 2021). An important caveat regarding research on immunesenescence is that most studies have been performed on peripheral blood. A few murine studies have reported an age‐related dysregulation; including a reduction in the size of Peyer's patches (PPs) and intestinal antigen‐specific SIgA antibody responses, resulting in an overall decline in mucosal immunity (Schmuker et al. 2001). As a consequence of ageing, we see increased susceptibility to infectious diseases particularly those affecting the gastrointestinal tracts, which is associated with increased risk of mortality. We hypothesise that the ageing host's gut microbiome composition influences the immune compartment which has received limited attention to date.

One of the key challenges in this area is that traditional techniques used to quantify age‐associated changes in gene expression, so far, have been spatially agnostic, limiting the interpretability of the findings. To address this gap, the aim of this study was to apply advanced digital spatial profiling technology to characterise the molecular and cellular changes associated with ageing. This approach seeks to improve our understanding of how the loss of tissue homeostasis drives age‐associated transformations in the complex interactions between the mucosa and immune microenvironment.

**FIGURE 1:**
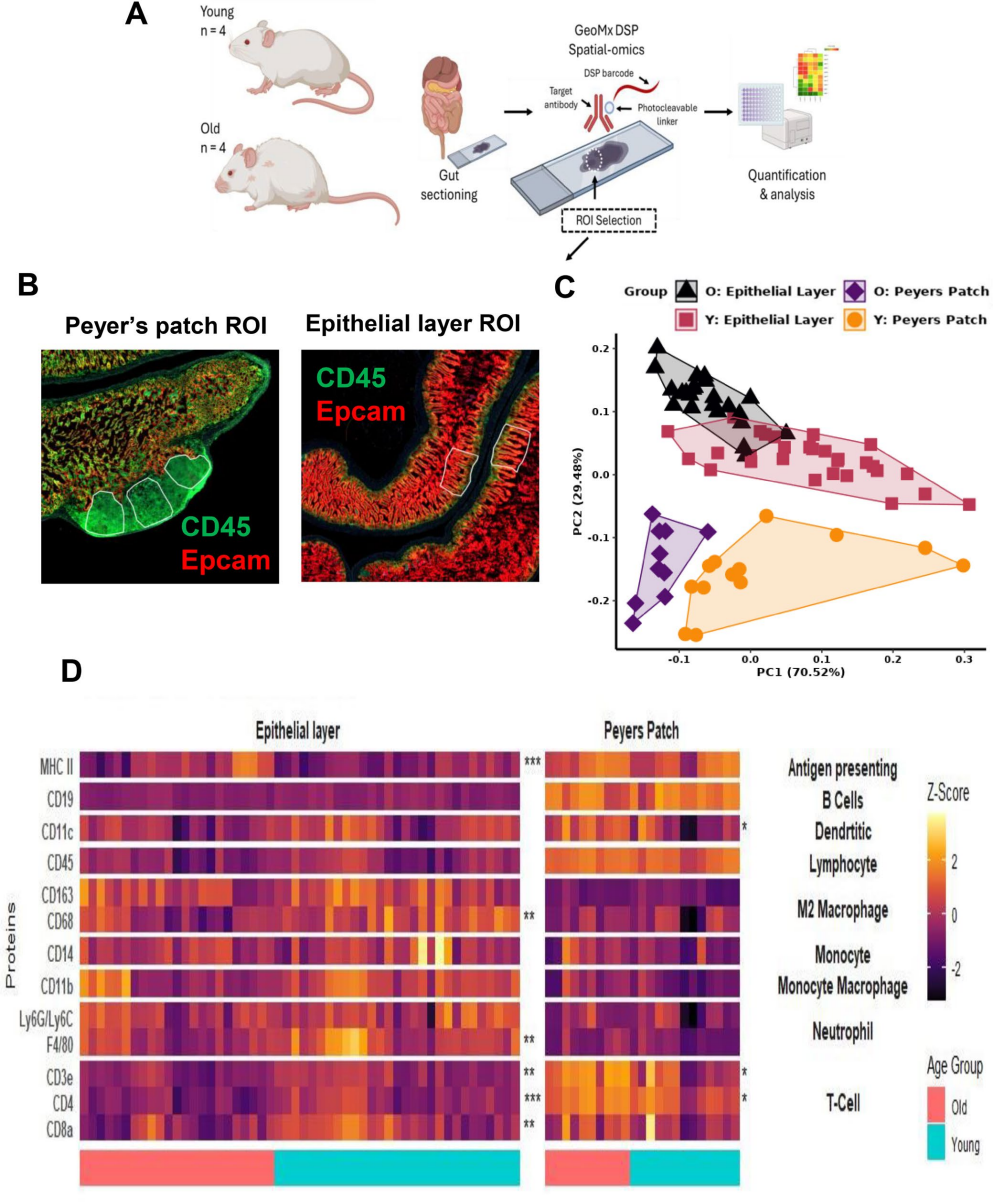
Spatial proteomics reveals differences in immune cell populations between tissue types. (A) An illustration of the workflow used to generate the spatial proteomics data. (B) Representative images of the ROIs, Peyer's patches and epithelial layer selected for GeoMx platform analysis. (C) Principal Component Analysis (PCA)‐based on protein expression levels of all 50 markers in the spatial proteomics panel separated by group and tissue type (Y: Young, O: Old). (D) Cell‐subset distribution was inferred from protein‐level expression of lineage‐specific markers between the two different regions (Peyer's patch and Epithelial layer) were compared separately in young and aged *C57BL/6* mice. Expression values represent normalised fluorescence intensity derived from the spatial proteomics platform (arbitrary fluorescence units), FDR corrected *p* value significance: ‘***’ 0.001, ‘**’ 0.01, ‘*’ 0.05.

To do so, colonic ileum biopsies were collected from young (2 months, *n* = 4) and aged (18–20 months, *n* = 4; 2 male 2 female) C57BL/6 mice. Following cryo‐sectioning, samples were stained with DAPI, and fluorochrome‐conjugated antibodies that target CD45 and Epcam to identify the epithelial cells and lymphocyte populations to pre‐determine the two compartments of interest and selection of Regions of Interest (ROI). Sixteen and 12 ROIs were selected in Peyer's patches from young and aged mice, respectively, and 29 and 28 ROIs from the epithelial layer of young and aged mice, respectively, to generate expression data for a panel of 50 proteins (Figure 1A–C). Four Nanostring GeoMx modules were employed, and expression levels were gathered for each ROI and quantified using NGS. Downstream analysis was performed using the inbuilt *mixedModelDE* function from the GeomxTools (version 3.18) to test for differential expression between groups independently for each tissue region and analysis pipeline in Rstudio (Figure 1). The Supporting Information provides detailed Methods S1.

### 1.1 Compartment Specific Differences in Immune Expression Profiles Between Young and Aged Mice

Firstly, we compared expression levels of proteins relevant for immune cell populations across two mucosal tissue compartments and although the total lymphocyte (CD45) expression remained unaltered with advancing age we observed a higher expression level of CD11c, a cell surface marker of antigen‐presenting dendritic cells (*p* = 0.007), CD3 a marker of T cells (*p* = 0.01), alongside CD4 T cells (*p* = 0.04) in the aged Peyer's patches (PP) (Figure 1C,D; Table S1), which has been previously reported by another study in aged mice (Santiago et al. 2011). On the contrary, we observed age‐associated loss in expression of CD3 density (*p* = 0.006), both in CD4 (*p* = 0.007) and CD8 expression (*p* = 0.001) in the ROIs of aged epithelial layer. We have also observed a decline in expression of F4/80 (*p* = 0.002), a well‐known marker for murine tissue‐resident macrophages, accompanied by a decline in MHC class II used by these cells to present antigens to CD4 T cells in an area requiring constant surveillance, which has been reported by another study driven by the pro‐inflammatory ageing microenvironment (Niess and Reinecker 2006; Figure 1D; Table S1).

### 1.2 Spatial Dissection of Age‐Related Proteins in Murine Gastrointestinal Tract With Advancing Age

**FIGURE 2:**
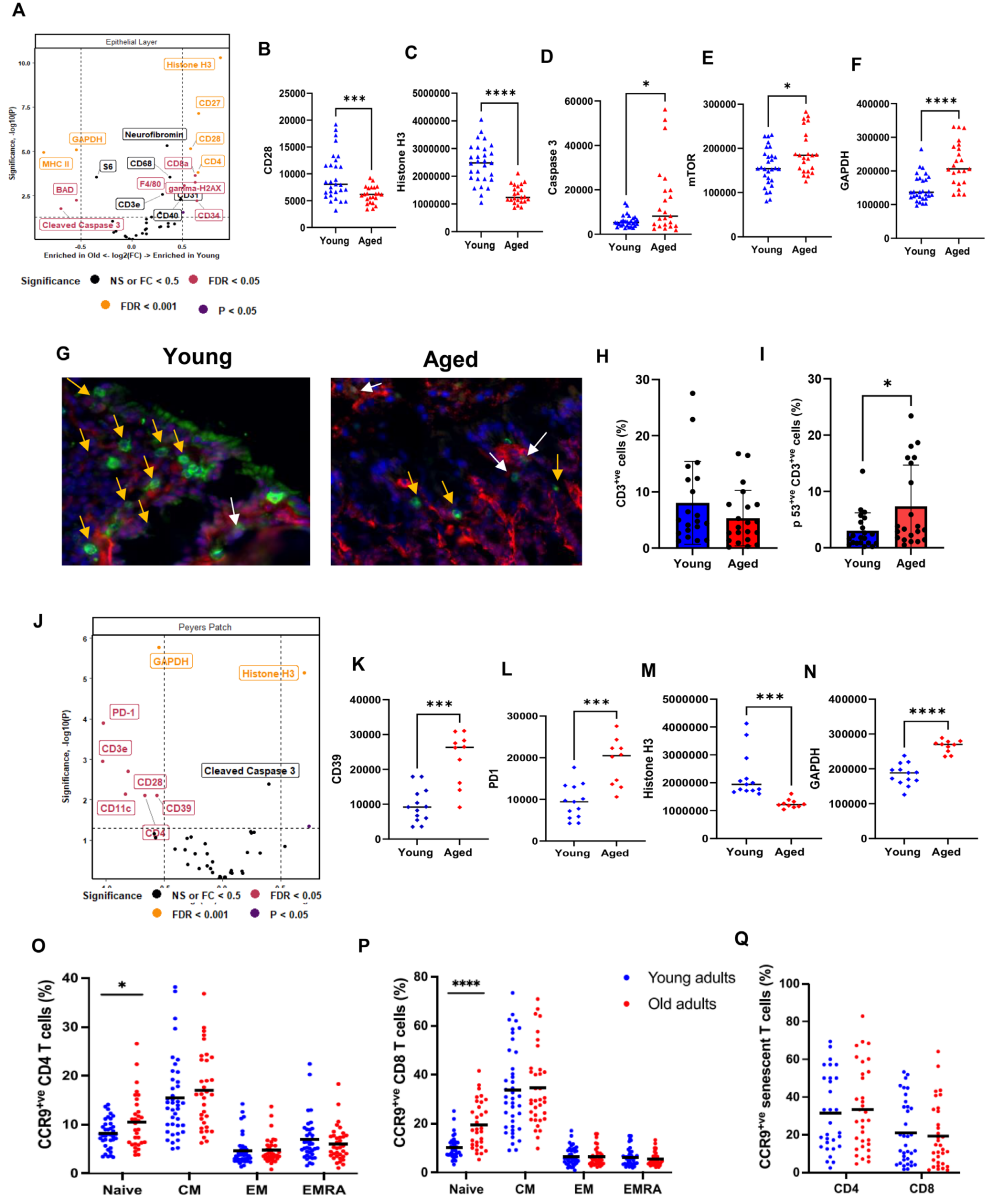
Enrichment of proteins in the gut of young and aged mice. The difference in expression of proteins in young and aged mice was compared using a volcano plot. Multiple different proteins were differentially expressed between the groups and in the epithelial layer (A) and Peyer's patch (J) regions. Panels (B–F) show differential protein expression between young and aged mice within the epithelial layer and panels (K–N) show differential protein expression between young and aged mice within the Peyer's patch region. Each dot represents a single region of interest (ROI) that passed all GeoMx spatial proteomics QC thresholds. Proteins were highlighted based on the significance of difference in expression after adjusting for FDR and fold change. FDR adjustments were made using the Benjamini–Hochberg procedure (G) Immunohistochemistry staining of the ileum from young and aged mice with DAPI (nuclear stain), CD3 (green), and p 53 (red) merged images with orange arrows showing CD3 single positive T cells and white arrows showing CD3 and p 53 double positive cells × 100 magnification. (H, I) Graph representing the number of CD3 and p 53 double positive cells per region of interest in young (*n* = 21) and aged (*n* = 18) C57BL/6J mice. Statistical analysis was performed by a two‐tailed Student's *t*‐test. The percentage of naïve, CM, EM and EMRA CD4 (O) and CD8 (P) T cells expressing CCR9. (Q) The frequency of CCR9‐positive senescent CD4 and CD8 T cells. Statistical analysis was performed by a two‐tailed Student's *t*‐test. ***0.001, **0.01, *0.05.

In the gut epithelial layer, 13 out of 45 age‐related proteins that were measured are differentially expressed between the young and aged gut epithelial layer but only 7 were differently expressed with age in PPs (Figure 2A). Firstly, we observed an age‐associated loss of CD34 (*p* = 0.01), a marker of intestinal stromal cells, known to play a crucial role in promoting maintenance of intestinal stem cells to repair damage (Wang et al. 2021), where its age‐associated loss could drive the loss of intestinal homeostasis (Table S2). Interestingly, we observed a reduced expression of co‐stimulatory molecule CD28 (*p* = 0.004) expressed primarily on T cells which is a well‐established marker of T cell senescence with the characteristic senescence‐associated secretory phenotype (SASP) of pro‐inflammatory cytokines and chemokines, contributing to inflammaging in older adults (Figure 2B). Importantly, we did not observe increased expression of canonical markers of senescence, such as p53 and p21 (Table S2) in this region; suggesting that senescence in this region is compartmentalised to T cells near the aged gut epithelium possibly due to repeated antigenic. It is technically challenging to validate spatial proteomic findings using qPCR or flow cytometry as both methods require homogenisation or dissociation of tissue, resulting in complete loss of spatial information, a limitation that is widely acknowledged in the spatial biology field. Thus, we performed immunofluorescence staining, consistent with approaches used in recent spatial studies and observed a trend towards a decrease in the total number of CD3^+^ T cells with age (*p* = 0.11), those that remain exhibit increased expression of senescence‐associated markers (*p* = 0.02). As no validated antibody for murine CD28 is available for tissue sections, we used p53 as a senescence‐associated marker for colocalisation with T cells (Figure 2G–I). Surprisingly, we observed higher levels of DNA damage (γH2Ax) marker in young gut epithelial tissue (*p* = 0.001), which might be reflective of a transient state of accumulation of genotoxic damage associated with high epithelial turnover in young tissue (Table S2). Furthermore, we observed a loss of heterochromatin stability, reflective in Histone H3 expression (*p* = 0.001) (Figure 2D). Age‐associated changes in gut microbiome composition and microbiome‐derived metabolite profile, including the loss of short chain fatty acids, such as butyrate that can induce epigenetic changes targeting histone modifications in enterocytes, thereby driving intestinal ageing (Joen et al. 2018). An upregulation of intrinsic apoptosis (programmed cell death) was observed in epithelial cells, identified by elevated levels of BAD (pro‐apoptotic member of Bcl‐2 family) known to promote mitochondria outer membrane permeabilization and activation of caspases 3 involved in cell death (Vogler et al. 2025) (*p* = 0.03; Figure 2E); hinting that the aged gut epithelial may avoid senescence but the loss of heterochromatin stability skews towards apoptotic clearance. Finally, we observed age‐related elevated expression of ribosomal protein S6 kinase (*p* = 0.01), a known mTOR (mammalian Target of Rapamycin) target that regulates T cell metabolism and accumulation of cellular damage; due to inhibition of autophagy (Figure 2F) and upregulation of key glycolytic enzyme GAPDH (glyceraldehyde‐3‐phosphate dehydrogenase) that has been associated with inflammation in the gut (*p* < 0.001) (Figure 2G).

Furthermore, on investigating changes in protein expressions in aged PPs, we have observed for the first time an upregulation of CD39, a marker expressed by activated T cells (*p* = 0.04), an observation which has been previously reported in systemic T cells of older adults (Fang et al. 2016) and PD‐1 (programmed cell death protein 1) expression (*p* = 0.002), a marker of exhausted cells (*p* = 0.002) (Figure 2I,J, respectively). Similar to the intestinal barrier region, we observed a loss of Histone H3 expression in aged PPs compared to young (*p* = 0.001) (Figure 2K) and upregulation of GAPDH (*p* < 0.001) (Figure 2L; Table S3).

### 1.3 Age‐Associated Changes in Gut‐Homing Dynamics of Blood T Cells of Older Adults

Our spatial proteomics revealed a pronounced accumulation of senescence‐associated T‐cell signatures in the aged mice intestinal tract, we next sought to determine whether senescent T cells are preferentially recruited from the circulation or whether naïve T cells home to the gut and acquire senescent features locally. Leukocyte trafficking to the intestinal tract is a tightly controlled process mediated by the chemokine receptor CCR9, which enables T cell homing by binding the chemokine ligand CCL25 expressed on IECs (Stenstad et al. 2006). In this study, we recruited forty healthy young and old individuals from whom systemic peripheral blood mononuclear cells (PBMCs) were isolated and CCR9 expression was assessed on T cell subsets via flow cytometry. Here, we observed a significant age‐associated increase in the frequency of naïve CD4 and CD8 T cells expressing CCR9 (*p* = 0.02 and *p* < 0.001 respectively) (Figure 2M,N). These aged naïve CD4 and CD8 T cells also had a greater CCR9 cell surface density (MFI) relative to those from young adults (Figure S1A,B). Furthermore, ageing did not affect the proportion of CD28^−ve^ senescent CD4 and CD8 T cells that expressed CCR9 (*p* = 0.68 for both) (Figure 2O); suggesting that the T cell senescence in the aged gut arises locally, possibly by repeated local antigenic stimulation of naïve T cells driving their senescence. This systemic signature complements the senescence‐related changes observed in mouse gut mucosa and supports a model in which T‐cell senescence precedes epithelial senescence and these cells may play a role in age‐associated loss of gut barrier dysfunction, but the precise contributions remain to be elucidated in future studies. However, questions on the ontogeny of these CCR9‐expressing T cells and the mechanisms governing their intestinal homing remain to be fully elucidated in future studies.

In summary, the application of novel spatial profiling techniques expands our understanding of drivers of mucosal ageing beyond previously described microbiome changes (Sovran et al. 2019), highlighting a potential role for the age‐associated accumulation of senescent T cells driven by repeated antigenic stimulation alongside an aged colonic epithelial layer that could further exacerbate mucosal inflammation which needs further investigation.

Future studies aimed at developing therapeutic strategies to promote healthy intestinal ageing hold promise for healthy ageing. For instance, inhibition of the mTOR pathway has been shown to increase H3 expression in epithelial cells and improve gut health by increasing intestinal autophagy, reducing intestinal stem cell (ISC) turnover and preventing age‐related gut leakiness (Zhang et al. 2024). Metformin, a widely prescribed antidiabetic drug with known mTOR inhibitory effects, has been reported to enhance the production of beneficial microbial products, reduce intestinal inflammation and increase expression of tight junction protein in mice (Wu et al. 2018). Targeting the elimination of senescent T cells using senolytic agents, such as Dasatinib and Quercitin (Zhu et al. 2015), or by microbiome‐derived metabolites such as butyrate (Rees‐Paddison et al. 2025) can alleviate the pro‐inflammatory phenotype of these cells and represents an alternative promising avenue for restoring intestinal homeostasis with age. Additionally, dietary interventions, including a low‐glucose diet or caloric restriction, can modulate the glycolytic flux through nutrient restriction to shift the metabolic profile of aged T cells that are on a metabolic overdrive (GAPDH upregulation) and can suppress mTOR, thereby directly reshaping immune–metabolic dynamics in the aged gut (Yilmaz et al. 2012; Wang and Green 2012). Together, the insights from this study lay the groundwork for targeted interventions that could delay or reverse age‐associated intestinal dysfunction, ultimately promoting healthier immune–epithelial crosstalk and improve outcomes in ageing populations.

## Author Contributions

N.A.D. gained funding for the study, participated in the design of the study, interpretation of the data and wrote the first draft of the manuscript. J.S. performed the data analysis for the spatial proteomics experiments and wrote the first draft of the manuscript. J.C. participated in sample processing and performed the immune phenotyping of human participants. M.C. gained funding for the animal experiments and participated in its experimental design. S.J.H. performed the animal experiments and collected the tissues. All authors edited and approved the final version of the manuscript.

## Funding

This work was supported by funding from the MRC‐Versus Arthritis Centre for Musculoskeletal Ageing Research. The funders provided financial support to this research but had no role in the design of the study, analysis, interpretation of the data and in writing the manuscript.

## Conflicts of Interest

The authors declare no conflicts of interest.

## Supporting information


**Table S1:** Expression levels of immune cell identification‐associated proteins in Peyer's patches and the intestinal epithelial layer of young versus aged mice Mean protein expression levels (± standard deviation) for all quantified proteins measured across ROIs in young and aged mice. Statistical analysis was performed by a two‐tailed Student's *t*‐test.
**Table S2:** Ageing‐associated changes in protein expression within the epithelial layer between young and aged mice. Mean protein expression levels (± standard deviation) for all quantified proteins measured across ROIs in young and aged mice. Statistical analysis was performed by a two‐tailed Student's *t*‐test.
**Table S3:** Ageing‐associated changes in protein expression within the Peyer's patches between young and aged mice. Mean protein expression levels (± standard deviation) for all quantified proteins measured across ROIs in young and aged mice. Statistical analysis was performed by a two‐tailed Student's *t*‐test.
**Figure S1:** CCR9 expression levels in human T cell subsets The CCR9 expression levels in naïve, CM, EM and EMRA CD4 (A) and CD8 (B) T cells expressing CCR9. (C) The expression levels of CCR9 in positive senescent CD4 and CD8 T cells. Multiple Unpaired Student's *T* tests and Mann–Whitney *U* tests were used to determine statistical differences between young (blue, *n* = 40) and old (red, 40) adults. *p ≤ 0.05, **p ≤ 0.001.

**Data S1:** acel70605‐sup‐0002‐DataS1.docx.

## Data Availability

The data that support the findings of this study are available on request from the corresponding author. The data are not publicly available due to privacy or ethical restrictions.
