# Supplementary material for: A Gut‐Centric View of Ageing: A Pilot Analysis Mapping Age‐Associated Immune and Molecular Alterations in Colonic Mucosa Using Spatial Proteomics
Source: Aging Cell. 2026 Jun 29;25(7):e70605. doi: 10.1111/acel.70605 (PMC13314716; doi:10.1111/acel.70605)
Supplement: Supplementary file 1 — Table S1: Expression levels of immune cell identification‐associated proteins in Peyer's patches and the intestinal epithelial layer of young versus aged mice Mean protein expression levels (± standard deviation) for all quantified proteins measured across ROIs in young and aged mice. Statistical analysis was performed by a two‐tailed Student's t‐test. Table S2: Ageing‐associated changes in protein expression within the epithelial layer between young and aged mice. Mean protein expression levels (± standard deviation) for all quantified proteins measured across ROIs in young and aged mice. Statistical analysis was performed by a two‐tailed Student's t‐test. Table S3: Ageing‐associated changes in protein expression within the Peyer's patches between young and aged mice. Mean protein expression levels (± standard deviation) for all quantified proteins measured across ROIs in young and aged mice. Statistical analysis was performed by a two‐tailed Student's t‐test. Figure S1: CCR9 expression levels in human T cell subsets The CCR9 expression levels in naïve, CM, EM and EMRA CD4 (A) and CD8 (B) T cells expressing CCR9. (C) The expression levels of CCR9 in positive senescent CD4 and CD8 T cells. Multiple Unpaired Student's T tests and Mann–Whitney U tests were used to determine statistical differences between young (blue, n = 40) and old (red, 40) adults. *p ≤ 0.05, **p ≤ 0.001. [file ACEL-25-e70605-s001.pdf]

	Young	Aged	P value
**Peyer's Patch**			
Ly6G/Ly6C	14271.8 (SD: 3654.26)	14969.28 (SD: 2753.73)	0.89
F4/80	6615.4 (SD: 2067.11)	7934.19 (SD: 2851.63)	0.39
CD14	32356.31 (SD: 8513.89)	40814.06 (SD: 13874.57)	0.23
CD11b	93076.06 (SD: 49766.19)	148804.79 (SD: 92424.79)	0.22
CD163	2612.97 (SD: 801.76)	2467.68 (SD: 636.09)	0.80
CD68	16050.86 (SD: 5215.65)	16228.62 (SD: 2795.93)	0.89
CD11c	127477.07 (SD: 86442.44)	215751.55 (SD: 73557.06)	**0.007**
MHC II	227303.38 (SD: 135764.75)	271054.83 (SD: 110677.6)	0.37
CD45	1151649.54 (SD: 469155.32)	1399815.01 (SD: 310336.85)	0.23
CD3e	11921.62 (SD: 9830.02)	23251.12 (SD: 7578.11)	**0.01**
CD4	43195.41 (SD: 22048.21)	70926.14 (SD: 23965.32)	**0.04**
CD8a	12764.35 (SD: 15832.82)	14305.13 (SD: 8018.24)	0.69
CD19	143525.29 (SD: 74440.41)	131784.75 (SD: 74687.01)	0.86
**Epithelial layer**			
Ly6G/Ly6C	18455.68 (SD: 3758.41)	17748.06 (SD: 3098.46)	0.93
F4/80	18343.09 (SD: 11539.29)	11167.05 (SD: 4302)	**0.002**
CD14	48385.07 (SD: 24148.73)	41891.79 (SD: 7521.22)	0.29
CD11b	304070.26 (SD: 144491.62)	262225.68 (SD: 253661.43)	0.06
CD163	9029.21 (SD: 5196.66)	7872.09 (SD: 5063.92)	0.24
CD68	22860.27 (SD: 5626.94)	17576.25 (SD: 4485.48)	0.11
CD11c	157246.49 (SD: 61610.65)	119134.89 (SD: 39653.8)	0.06
MHC II	64698.78 (SD: 30899.83)	128386.41 (SD: 107886.58)	**9.06E-05**
CD45	283962.61 (SD: 175449.32)	203385.44 (SD: 115645.58)	0.17
CD3e	7557.39 (SD: 2374.13)	5955.02 (SD: 1814.33)	**0.006**
CD4	18848.22 (SD: 10029.45)	11176.54 (SD: 4407.11)	**0.007**
CD8a	12490.54 (SD: 5777.78)	8044.41 (SD: 4658.23)	**0.001**
CD19	14795.64 (SD: 4185.73)	12902.42 (SD: 2857.57)	0.09

Supplementary Table 1

	Young	Aged	P value
**Epithelial layer**			
BAD	280033.97 (SD: 170321.62)	409321.95 (SD: 236258.03)	**0.01**
BatF3	28963.07 (SD: 6814.71)	26892.27 (SD: 5271.18)	0.63
Bclxl	173035.68 (SD: 61043.09)	171353.69 (SD: 84709.92)	0.54
BIM	10642.89 (SD: 3645.69)	10136.93 (SD: 3847.62)	0.98
CD27	6677.51 (SD: 2584.09)	4289.46 (SD: 1261.74)	**1.71E-06**
CD31	122608.25 (SD: 41514.54)	95501.43 (SD: 52854.1)	0.11
CD34			**0.01**
CD39	73864.03 (SD: 41233.18)	55626.4 (SD: 23474)	0.24
CD40	13624.09 (SD: 5435.06)	9825.38 (SD: 4358.45)	0.11
CD40L	20100.6 (SD: 15769.94)	16166.59 (SD: 4442.52)	0.49
CD44	290595 (SD: 169529.43)	256935.86 (SD: 194862.82)	0.24
CD86	8649.44 (SD: 1992.42)	7657.18 (SD: 1605.89)	0.17
CD127	122835.6 (SD: 61882.03)	105326.79 (SD: 35109.91)	0.72
Fibronectin	49027.4 (SD: 28285.74)	41909.96 (SD: 33582.04)	0.18
GZMB	108054.81 (SD: 38755.26)	126633.66 (SD: 75887.35)	0.40
gamma H_2Ax	33688.4 (SD: 16496.59)	20502.75 (SD: 7839.2)	**0.001**
Neurofibromin	47937.67 (SD: 7873.12)	37639.42 (SD: 9916.04)	0.96
Ki-67	90972.26 (SD: 69318.03)	82389.98 (SD: 39103.39)	0.84
p 21	9143.32 (SD: 2650.82)	9751.21 (SD: 4482.71)	0.66
p 53	11902.24 (SD: 3402.74)	11918.34 (SD: 5041.3)	0.98
PARP	13276.23 (SD: 4618.24)	13817.54 (SD: 3009.04)	0.14
PD1	3132.7 (SD: 1042.8)	2906.48 (SD: 1021.23)	0.34
PDL1	19693.59 (SD: 6663.12)	19253.23 (SD: 5379.23)	0.89
Perforin	14657.21 (SD: 3038.76)	13377.15 (SD: 3295.95)	0.11

Supplementary Table 2

	Young	Aged	P value
**Peyer's Patch**			
BAD	82579.8 (SD: 30892.32)	69398.27 (SD: 15154.09)	0.22
BatF3	17635.42 (SD: 5357.36)	16814.97 (SD: 1212.03)	0.80
Bclxl	25517.22 (SD: 13182.69)	26197.48 (SD: 6351.44)	0.91
BIM	18950.62 (SD: 7822.56)	21074.27(SD: 5044.86)	0.76
Caspase 3	3364.46 (SD: 940.05)	2534.52 (SD: 497.49)	n.s
CD27	11118.02 (SD: 6002.03)	12899.29 (SD: 2686.84)	0.37
CD31	52641.93 (SD: 21463.75)	58102.31 (SD: 19254.71)	0.80
CD34	23339.98 (SD: 7802.47)	20319.29 (SD: 4170.79)	0.22
CD40	67716.86 (SD: 32800.85)	73216.94 (SD: 31409.37)	0.76
CD40L	10163.98 (SD: 2559.78)	11513.56 (SD: 2527.86)	0.61
CD44	274310.2 (SD: 136221.86)	382265.69 (SD: 113252.85)	0.23
CD86	12180.14 (SD: 3419.34)	13475.6 (SD: 3997.5)	0.71
CD127	49611.67 (SD: 20673.34)	46113.17 (SD: 5419.47)	0.80
Fibronectin	17967.95 (SD: 12545.32)	12069.58 (SD: 8700.38)	0.33
GAPDH	184008.13 (SD: 30804.68)	264692.93 (SD: 18108.02)	**8.01E-05**
GZMB	35303.06 (SD: 10021.36)	36641.83 (SD: 4191.04)	0.89
gamma H_2Ax	85599.44 (SD: 45146.65)	62373.1 (SD: 15021.49)	0.39
Histone H3	2241051.55 (SD: 819566.76)	1252980.96 (SD: 158847.2)	**0.001**
Neurofibromin	45445.22 (SD: 17881.27)	38900.7 (SD: 6901.66)	0.39
Ki-67	183582.01 (SD: 160009.79)	89373.75 (SD: 28853.27)	0.21
p 21	7702.96 (SD: 2482.39)	8143.65 (SD: 1819.6)	0.89
p 53	6623.79 (SD: 1517.44)	5803.48 (SD: 1236.3)	0.22
PARP	40245.76 (SD: 18660.53)	33974.58 (SD: 14788.28)	0.86
PDL1	27484.42 (SD: 9810.67)	27311.79 (SD: 5133.15)	0.96
Perforin	11558.36 (SD: 3096.33)	13109.2 (SD: 1432.78)	0.30
S6 kinase	138368.26 (SD: 41177.88)	155877.04 (SD: 18068.88)	0.39

Supplementary Table 3

	**Young**	**Aged**	**P value**
**Peyer's Patch**			
CD28	14275.25(SD: 6471.79)	25424.52(SD: 8263.46)	0.01921
CD39	19659.17(SD: 7590.6)	28513.16(SD: 6969.73)	0.041432
FOXP3	1554.5(SD: 517.57)	1549.65(SD: 272.6)	0.806191
PD1	9390.93(SD: 4125.47)	18931.98(SD: 5607.86)	0.002028
**Epithelial layer**			
CD28	9447.12 (SD: 4325.68)	6061.3 (SD: 1574.56)	7.6661e-05
CD34	96382.04 (SD: 75051.66)	53825.85 (SD: 20712.24)	0.012586
Caspase 3	6928.22 (SD: 6151.23)	15409.41 (SD: 16243.5)	0.03354
FOXP3	1786.97 (SD: 912.81)	1332.82 (SD: 220.99)	0.004031
GAPDH	147790.06 (SD: 40152.85)	220015.51(SD: 64494.78)	7.67E-05
Histone.H3	2454472.28 (SD: 719802.65)	1337451.82 (SD: 331069.07)	2.42E-09
S6 kinase	157636.62 (SD: 42545.03)	191336.08 (SD: 48896.68)	0.001134

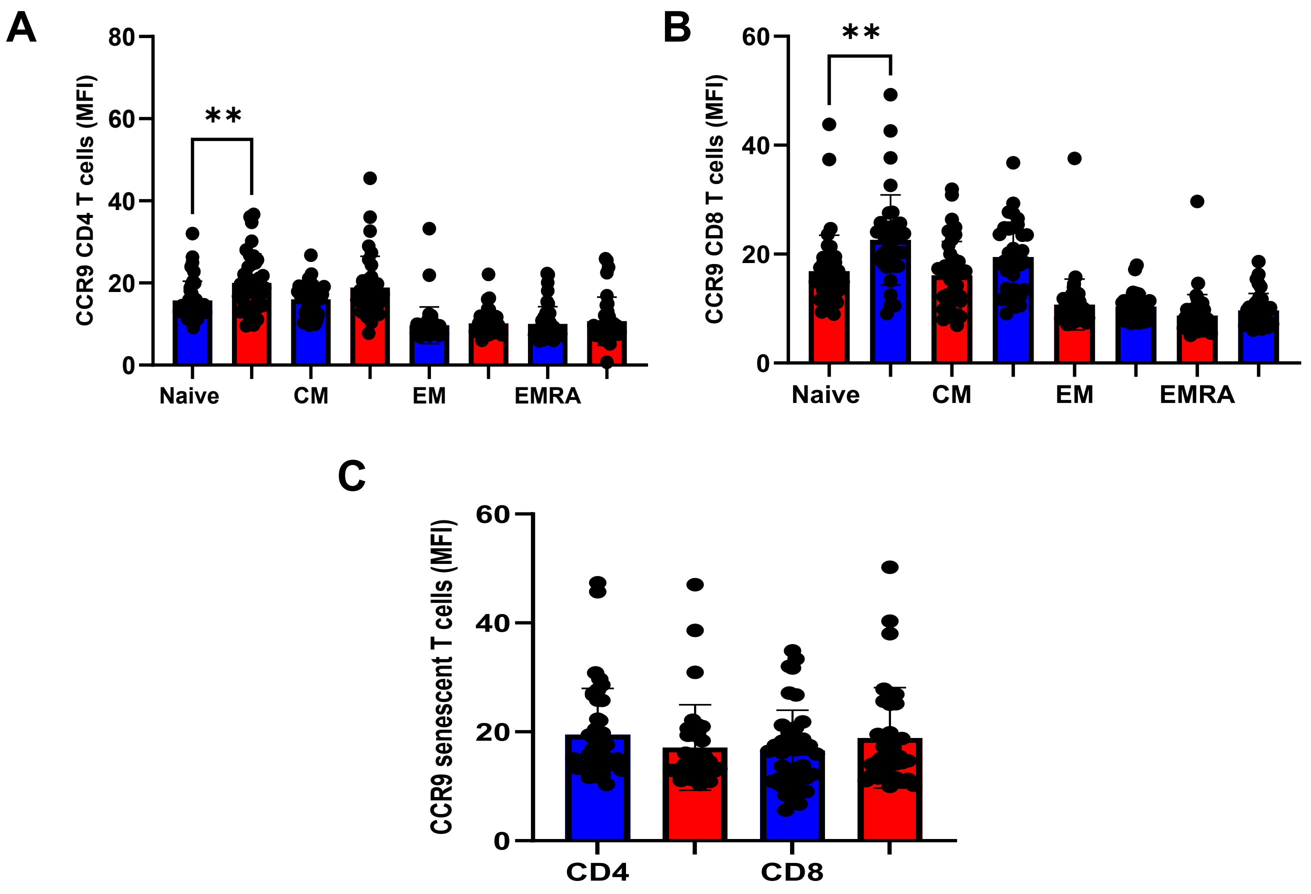

Supplementary Figure 1 :CCR9 expression levels in human T cell subsets
The CCR9 expression levels in naïve, CM, EM and EMRA CD4 (A) and CD8 (B) T cells expressing CCR9. (C) The expression levels of CCR9 in positive senescent CD4 and CD8 T cells. Multiple Unpaired Student's T tests and Mann-Whitney U tests were used to determine statistical differences between young (blue, n = 40) and old (red, 40) adults. *p ≤0.05, **p ≤ 0.001.
