# Supplementary material for: A Gut‐Centric View of Ageing: A Pilot Analysis Mapping Age‐Associated Immune and Molecular Alterations in Colonic Mucosa Using Spatial Proteomics
Source: Aging Cell. 2026 Jun 29;25(7):e70605. doi: 10.1111/acel.70605 (PMC13314716; doi:10.1111/acel.70605)
Supplement: Supplementary file 2 — Data S1: acel70605‐sup‐0002‐DataS1.docx. [file ACEL-25-e70605-s002.docx]

**Methods S1**

Spatial

Animal studies were regulated by the Animals (Scientific Procedures) Act 1986 of the United Kingdom and performed under Personal Project Licence (PE5985209) at the Biomedical Services Unit, University of Birmingham, which holds a section 2C Establishment Licence. Approval was granted by the University of Birmingham’s Animal Welfare and Ethical Review Body, and all ethical guidelines were adhered to whilst carrying out this study.Wild-type young (2 months, n = 4) and aged (18-20 months, n = 4) female and male C57BL/6J mice were purchased (The Jackson Laboratory, USA) and housed in the University of Birmingham Central Animal Facility prior to experimentation. Gastrointestinal tracts were separated into small and large intestines by making two cuts: one above the stomach and the other just above the caecum (Figure 2.8A). After discarding the stomachs, the faecal content was expelled from mouse gastrointestinal tracts, and the surrounding adipose tissue was carefully removed. The small and large intestines were then carefully coiled in separate plastic Tissue-Tek® cryomold moulds (15x15x5 mm) and mounted in TissueTek® OCT compound. OCT-embedded tissues were snap frozen on dry ice and stored at -80°C prior to cryosectioning.

Cryosectioning was carried out in a Leica CM1950 Cryostat (Leica Biosystems, Germany) with a chamber and block temperature of -20°C at the University of Birmingham. Tissue sections were cut randomly throughout the gut at a thickness of 7 μm. Two tissue sections (7 μm thick) per age group were mounted onto a slide. All slides were stored at -80°C before immunostaining with CD45 (clone: EM-05; Novus Bio. Diluted 1:100. AF647-conjugated), Epcam (clone: G8.8 BioLegend. Diluted 1:800. AF488-conjugated) and a nuclear stain (DAPI) to identify the epithelial cells and lymphocyte populations that were used to pre-determine the two compartments of interest and regions of Interest (ROI) to generate expression data for a panel of proteins. Regions were chosen by manually drawing in the two compartments (Epithelial layer & Peyer’s patches) from each young and aged mouse [Figure 1]. Spatial profiling was undertaken on the NanoString GeoMx™ Digital Spatial Profiling platform at the Birmingham Tissue Analytics at University of Birmingham. Four Nanostring GeoMx modules were used in addition to the “Core” module (Cell Death, Immune Activation Status, Immune Cell Typing, Myeloid) and expression levels were gathered for each ROI as per the GeoMx DSP protocol that was sequenced on an Illumina NextSeq 550. Downstream analysis was performed using the GeomxTools (version 3.18) package and analysis pipeline in Rstudio (version 4.3.0). The proteins (GFP, FOXP3 and ICOS) were removed due to low signal to background ratio and no points of expression exceeding background (three negative control targets) or housekeeping protein expression. Normalisation was performed using housekeeping proteins.

Data analysis

Raw data (DCC, PKC and OME.TIFF files) were exported from GeoMx DSP for downstream analysis in R Studio (Version 4.3.0). Thirteen regions of interest failed segment QC checks due to low saturation (<40%). Normalisation was performed using the housekeeping gene method (hk_norm) with very low total counts were removed from analysis. Counts were summarized to the gene level by summing passed probes for each gene, using a filtering method similar to ‘Biological Probe QC’ as described in the GeoMx NGS Data analysis user manual (v2.0). Specifically, if the geomean of a probe across all samples is less than 10% of the sum for all probes for a given gene (low counts) it is excluded. Or, if it fails a Grubbs outlier test (one sided up or down, p-value <0.01) in more than 20% of samples. No gene-level filtering was performed.

Tissue sectioning and staining protocol:

Pre-frozen OCT tissue was sectioned using a Leica CM 1950 Cryostat at a thickness of 7µm. Sections were brought to temperature and washed with PBS prior to staining. After washing sectioned were treated with a mixture of primary antibodies P53 (80077-1-RR, Proteintech) and CD3 (100201, Biolegend) at a 1:500 dilution in PBS containing 10% FCS for blocking, 0.2% triton-x for permeabilisation, and incubated at 4ºC for 24 hours. Samples were then washed with PBS prior to the addition of secondary antibodies (A32732, Invitrogen; A11006, Invitrogen) at a dilution factor of 1:300 in PBS with 0.2% triton-x and incubated at room temperature for 1 hour. Slides were then washed and mounted using Vectashield PLUS antifade mounting medium (H-1900, Vector Laboratories) which contains DAPI.

Fluorescence image analysis:

Image analysis was performed using ImageJ (Version 1.54g). Individual cells we mapped using DAPI images, they were converted to 8-bit images, before being “thresholded”, this primary binary mask was mathematically dilated to generate whole-cell regions of interest (ROIs). “Watershed” was applied to separate closely clustered ROIs. Valid cells were then defined using the “analyse particles” tool with a size exclusion criteria set to >50 pixels. These regions of interest were then applied to the raw, unedited subsequent marker images (CD3, P53) to extract the mean intensity per cell. For each image, the background staining was established by measuring a negative region of the tissue, and the “positive” cut off was defined as the background mean + 3 standard deviations.  Cells were classed as positive based on these image specific thresholds. Multiple images (minimum of 10) per-biological replicate was analysed per animal.

Human Population

Ethical approval for this study was granted by the HRA and Health and Care Research Wales (HRCW) Approval (IRAS 301974) and written consent was obtained from individuals prior to participation. Older participants had to be >65 years of age (n = 40) and younger participants (n = 40) had to be between 18-35 years of age. All participants were required to be in good health and donated blood samples collected into heparinized vacutainers® (BD Biosciences, USA).

Peripheral blood mononuclear cells (PBMC) isolation.

The collected blood samples were pooled from heparinised vacutainers into 25 ml universal tubes (Scientific Laboratory Supplies, UK) and diluted with RPMI-1640 media supplemented with glutamine, penicillin and streptomycin (Life Technologies Limited, UK) at a 1:1 ratio. The blood-RPMI mix was layered on top of 6 ml of Ficoll-Plaque^TM^ Plus (GE Healthcare, UK) and centrifuged at 400 × g for 30 minutes at room temperature (RT) with no break or acceleration. Post centrifugation, PBMCs were transferred into a fresh universal tube containing MACS running buffer (Miltenyi Biotec, UK). Once all PBMCs were transferred, the universal tube was topped up with autoMACS running buffer and centrifuged at 300 × g for 10 mins at RT with full brake and acceleration. Post spin, the pellet was resuspended in 25 ml of autoMACS running buffer and washed via centrifugation at 300 × g for 10 minutes at RT. 1 ml of a prepared freezing solution containing 5 ml of 10% DMSO (Sigma Alrich, UK) and 45 ml of heat-inactivated FCS (Thermofisher, UK) was used to resuspend the pellet. 500 µl of PBMCs were aliquoted into 1 ml cryovials and then placed in a Mr Frosty^TM^ Freezing Container (ThermoFisher, UK) in which the cells were frozen at -80°C.

Immunostaining via Flow cytometry

Post thawing of frozen PBMCs were stained with a combination of antibodies to assess the frequence of gut homing marker (CCR9) in the CD4^+^ and CD8^+^ T cells subsets defined as naïve (CD45RA^+^CCR7^+^), central memory (CD45RA^-^CCR7^+^), effector memory (CD45RA^-^CCR7^-^ and terminally differentiated effector memory cells (CD45RA^+^CCR7^-^) and senescent (CD28^-^). Frequency of CCR9 expressing cells and expression levels were assessed in each T cell subset using a MACSQuant® Analyzer 8 flow cytometer (Miltenyi Biotec, Germany) and data was analyzed using FlowJo software (FlowJo LLC, USA). The key antibodies are listed below (S2).

Statistical analysis

A p value of <0.05 was deemed to be significant. FDR adjustments were made using the Benjamini-Hochberg procedure. FDR corrected p value significance: ‘***’ 0.001, ‘**’ 0.01, ‘*’ 0.05. To ensure that all figures are readable for the greatest number of people all colour figures were generated using the Viridis colour map R package (Garnier et al., 2023), which was developed to improve the readability of visual data for those who may otherwise have issues viewing them. Graphs were created using GraphPad PRISM software v10 (GraphPad software, USA).

**S2 Methods**

**List of antibodies used in flow cytometry**

| **Antibody** | **Supplier** | **Clone** |
| --- | --- | --- |
| Anti-human CD3-PEcy7 | Thermo Fisher | UCHT1 |
| Anti-human CD4 Violet | Thermo Fisher | RPA-T4 |
| Anti-human CD8 PE cy5.5 | Immunotools | UCHT4 |
| Anti-human CCR7 FITC | R and D systems | 150503 |
| Anti-human CD45RA APC | Biolegend | HI-100 |
| Anti-human CCR9 PE | R and D systems | MAB179 |
| Anti-human CD28 APC | B D Biosciences | CD28.2 |
| Anti-human CD57 FITC | Thermo Fisher | HCD57 |
| Anti-human CD80 FITC | BD Biosciences | L307.4 |
| Anti-human CD86 PE | BD Biosciences | 2331 |
| **Transcription factors** |  |  |
| Anti-human Foxp3 PE | Thermo Fisher | PCH101 |
| Anti-human bcl6 APC | Thermo Fisher | BCL-UP |
| **Isotype controls** |  |  |
| Mouse IgG1 PE | Biolegend | MPC11 |
| Mouse IgG1 FITC | Thermo Fisher | eBM2a |
| Mouse IgG2b Violet | Biolegend | MOPC21 |
| Mouse IgG1 APC | Biolegend | MOPC21 |
| Mouse IgG1PEcy7 | Thermo Fisher | P3.6.281 |
| Mouse IgG2b PEcy5.5 | Biolegend | MPC11 |
